# Chronic comorbid conditions and asthma exacerbation occurrence in a general population sample

**DOI:** 10.1038/s41533-023-00350-x

**Published:** 2023-08-11

**Authors:** Emma Baljet, Hilde Luijks, Lisette van den Bemt, Tjard R. Schermer

**Affiliations:** 1 Department of Primary and Community Care, Radboud Institute for Health Sciences, Radboud University Medical Center, Nijmegen, The Netherlands; 2 General Practice Valkenburg, Valkenburg, The Netherlands; 3 Science Office, Gelre Hospitals, Apeldoorn, The Netherlands

**Keywords:** Asthma, Respiratory signs and symptoms, Epidemiology

## Abstract

Chronic comorbid conditions are common in adults with asthma, and some may influence a patient’s asthma exacerbation risk. We explored associations between eighteen chronic comorbid conditions and asthma exacerbation occurrence in adults with asthma in a cross-sectional study nested within a cohort study using data from the two-yearly US National Health and Nutrition Examination Survey (NHANES) program. Data of 2387 adults with self-reported doctor-diagnosed current asthma from the 2007 to 2018 NHANES surveys were selected. Investigated chronic comorbidities were: angina pectoris; congestive heart failure; coronary heart disease; depression; diabetes mellitus; soft and hard drug use; gastroesophageal reflux; gout; history of heart attack; history of stroke; hypercholesterolemia; hypertension; kidney failure; liver conditions; obesity; rheumatoid arthritis; and thyroid problems. Outcome was defined as asthma exacerbation category: no, moderate, or severe exacerbation(s) in the past year. Ordinal logistic regression analysis with correction for potential confounders was used to estimate odds ratios (OR) for moderate or severe exacerbations. Observed associations with increased severe asthma exacerbation occurrence were: obesity (OR = 1.67; 95% confidence interval 1.24, 2.26), and rheumatoid arthritis (OR = 1.55; 1.04, 2.30). History of stroke (OR = 1.95; 1.22, 3.11) and rheumatoid arthritis (OR = 1.33; 1.00, 1.75) showed associations with increased moderate exacerbation occurrence. Age-stratified analysis showed soft drug use, obesity, depression, thyroid problems, and rheumatoid arthritis to be associated with moderate and/or severe exacerbation occurrence in one or more 10-year age strata. In conclusion, several chronic comorbid conditions were associated with asthma exacerbation occurrence, which confirms but also complements previous studies. Our observations contribute to understanding exacerbation risk estimation and, ultimately, personalized asthma management.

## Introduction

Asthma is a common chronic airways disease, with a prevalence of 8.4% among adults in the United States (US) in 2018^1^. The typical chronic airway inflammation is characterized by symptoms of airflow limitation that vary over time and in intensity. Patients with asthma may experience episodic flare-ups (acute exacerbations) of their asthma, which can be life-threatening^2^. Poor asthma control involves symptoms that can affect activities of daily living and increase the risk of undesirable outcomes, including exacerbations^2^. Uncontrolled asthma is a considerable cause of asthma morbidity, mortality, and healthcare costs^3^.

Presence of other chronic morbidities is common in adults with asthma^4^. In primary care an overall presence of one or more comorbid conditions in 72.8% in female and 65.3% in male asthma patients has been observed^5^. The current literature provides ambiguous results regarding possible associations between specific chronic comorbidities and increased risk of asthma exacerbations. Multiple studies found overweight and obesity to be associated with increased asthma exacerbation risk^6–8^. Another often studied chronic comorbid condition is gastroesophageal reflux disease (GERD), which is more common in severe asthma compared to mild-to-moderate asthma^9^. Several studies have reported an association between GERD and worse asthma control^6–8^, although a systematic review found no consistent benefit on asthma symptoms following treatment for gastroesophageal reflux^10^. Recent studies have shown a higher incidence of cardiovascular diseases (i.e., acute myocardial infarction and ischemic stroke) in patients with asthma^11,12^ and that lack of asthma control is associated with increased risk of stroke^13,14^. An association between rheumatoid arthritis and risk of asthma exacerbations has been reported^15^, whereas depression has shown to be relatively common in exacerbation-prone asthma^16^. This is explained by brain centers’ functional response to psychological disease and stress and the subsequent stress hormones production from the neuroendocrine system, leading to increased asthma exacerbation risk^17^.

More insight in the prevalence of chronic comorbid conditions in patients with asthma and, especially, a better understanding of their influence on asthma exacerbation occurrence and thus asthma control may contribute to patient-centered decision making, chronic disease management, and better asthma-related outcomes. Therefore, in this study we investigated (i) the prevalence of eighteen different chronic comorbid conditions and (ii) explored their associations with asthma exacerbation occurrence in a US general population sample of adults with a self-reported diagnosis of asthma. As no specific a priori hypotheses to be tested were formulated, the study should be considered to be hypotheses-generating in nature.

## Results

### Study population characteristics

Table 1 shows the baseline characteristics of the study population (*n* = 2387), which consisted for the larger part of females (65.1%) and non-Hispanic whites (40.9%). Among the study population 1344 subjects (56.3%) reported not to have had asthma exacerbations in the past year, 281 (11.8%) reported at least one severe exacerbation, and 762 (31.9%) reported one or more moderate but no severe exacerbations. Statistically significant differences between the three asthma exacerbation categories were observed for sex, race, PIR, and most categories of asthma medication (i.e., SABA, LABA, ICS, OCS, leukotriene modifiers, anticholinergics, and antihistamines). The proportion of subjects using anti-inflammatory medication (i.e., ICS, OCS and leukotriene modifiers) and bronchodilators (i.e., SABA, LABA, and anticholinergics) increased with the severity of the asthma exacerbation category. Age, time since asthma diagnosis, health insurance coverage, and smoking status were not statistically significant different between the exacerbation categories.

**Table 1:** Study population characteristics by asthma exacerbation category.

		Asthma exacerbation category			
	Total (*n* = 2387)	No exacerbations (*n* = 1344)	Moderate exacerbation(s) (*n* = 762)	Severe exacerbation(s) (*n* = 281)	*p*-value^c^
Age, mean (SD), years	44.9 (18.4)	45.5 (19.4)	44.5 (17.3)	43.3 (16.4)	0.328
Asthma duration, mean (SD), years	21.4 (16.2)	21.3 (16.4)	21.5 (16.0)	21.3 (16.0)	0.956
Sex					<0.001
Male	834 (34.9)	535 (39.8)	231 (30.3)	68 (24.2)	
Female	1,553 (65.1)	809 (60.2)	531 (69.7)	213 (75.8)	
Race and ethnicity					<0.001
Non-Hispanic White	977 (40.9)	542 (40.3)	353 (46.3)	82 (29.2)	
Non-Hispanic Black	668 (28.0)	384 (28.6)	180 (23.6)	104 (37.0)	
Mexican American	236 (9.9)	141 (10.5)	66 (8.7)	29 (10.3)	
Other	506 (21.2)	277 (20.6)	163 (21.4)	66 (23.5)	
Health insurance coverage					0.549
Yes	1,979 (82.9)	1,120 (83.3)	630 (82.7)	229 (81.5)	
No	404 (16.9)	220 (16.4)	132 (17.3)	52 (18.5)	
Unknown	4 (0.2)	4 (0.2)	—	—	
Poverty income ratio (PIR)^a^					0.004
>1	1,535 (64.3)	876 (65.2)	501 (65.7)	158 (56.2)	
≤1	622 (26.1)	334 (24.9)	192 (25.2)	96 (34.2)	
Unknown	230 (9.6)	134 (10.0)	69 (9.1)	27 (9.6)	
Cigarette smoking status					0.081
Never	1,374 (57.6)	792 (58.9)	426 (55.9)	156 (55.5)	
Former	515 (21.6)	298 (22.2)	163 (21.4)	54 (19.2)	
Current	498 (20.9)	254 (18.9)	173 (22.7)	71 (25.3)	
Asthma medication					
SABA	626 (26.2)	231 (17.2)	270 (35.4)	125 (44.5)	<0.001
LABA	277 (11.6)	126 (9.4)	107 (14.0)	44 (15.7)	<0.001
Anticholinergics	71 (3.0)	31 (2.3)	22 (2.9)	18 (6.4)	0.001
ICS	325 (13.6)	142 (10.6)	126 (16.5)	57 (20.3)	<0.001
OCS	67 (2.8)	22 (1.6)	25 (3.3)	20 (7.1)	<0.001
Leukotriene modifiers	209 (8.8)	106 (7.9)	68 (8.9)	35 (12.5)	0.047
Antihistamines	154 (6.5)	68 (5.1)	71 (9.3)	15 (5.3)	0.001
Other^b^	8 (0.3)	1 (0.1)	3 (0.3)	4 (1.4)	—

Figures are *n* (%) unless stated otherwise.

*ICS* inhaled corticosteroids, *LABA* long-acting β_2_-agonist, *OCS* oral corticosteroids, *SABA* short-acting β_2_-agonist.

^a^ PIR classified as income at or below the poverty line ( ≤1) or above the poverty line ( >1).

^b^ cromolyn, epinephrine, immunomodulator medications, phosphodiesterase-4 (PDE_4_) inhibitors, and unspecified anti-asthmatic combinations, -respiratory agents, and -respiratory inhalant products.

^c^ no correction for multiple comparisons was applied. Variables with counts below 8 in one or more asthma exacerbation categories were not statistically tested.

### Distribution of chronic comorbid conditions in different age categories

**Fig. 1:**
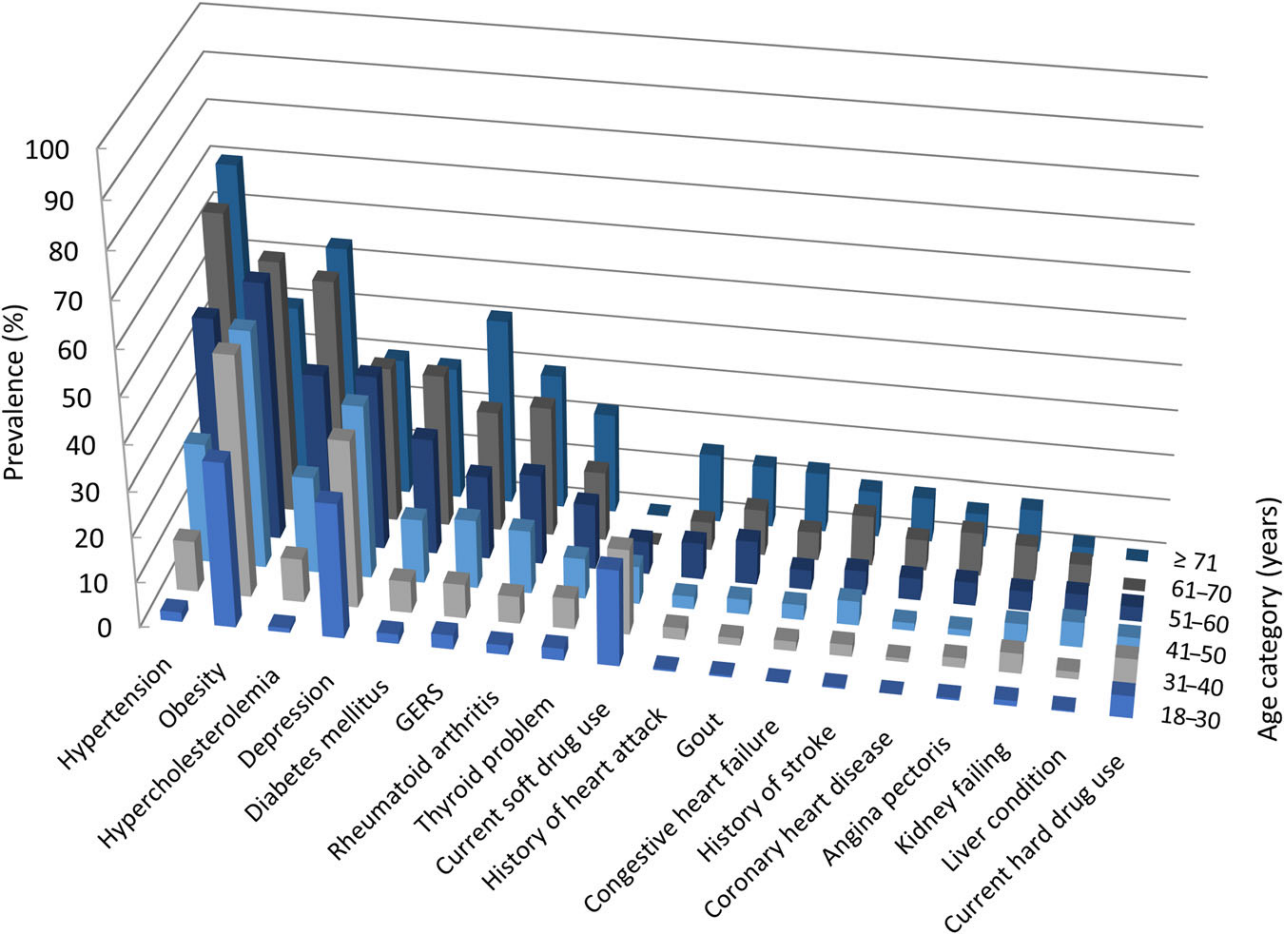
Prevalence rates of chronic comorbid conditions in different age categories of subjects with self-reported doctor-diagnosed asthma (*n* = 2387). The height of each bar represents the proportion (%) of all subjects in the respective age category suffering from the condition. GERS gastroesophageal reflux-like symptoms.

**Fig. 2:**
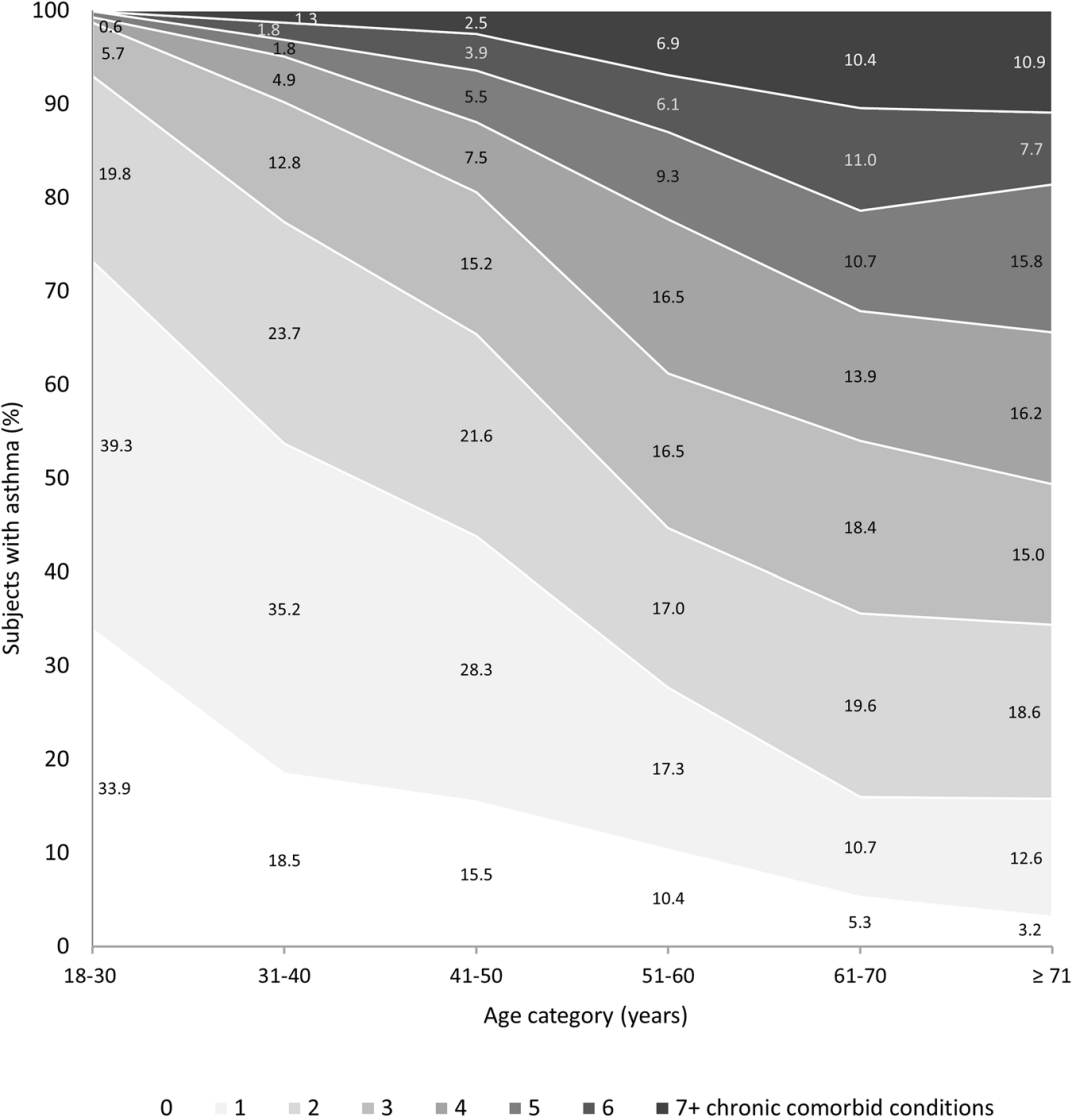
Frequency of the cumulative number of chronic comorbid conditions in the different age categories in subjects with a self-reported diagnosis of asthma (*n* = 2387). The shade of grey represents the total number of comorbid conditions, the exact percentages are shown in the figure.

Table 2 shows the prevalence of eighteen different chronic comorbid conditions in the total study population and in the three asthma exacerbation categories. Comorbid conditions with an overall prevalence >10% in the study population were obesity (49.6%), depression (34.1%), hypertension (30.3%), hypercholesterolemia (23.9%), diabetes mellitus (15.6%), rheumatoid arthritis (13.8%), and soft drug use (11.4%). Figure 1 shows the prevalence distribution of the chronic comorbid conditions in the different age categories. Prevalence of most conditions increased with age, except for soft drug use which showed a decreasing trend with age. Depression showed a high prevalence in all age categories, with rates around 30%. Cardiovascular conditions showed an increasing trend with age, with the highest prevalence in the ≥71 years age category for hypertension, hypercholesterolemia, congestive heart failure, coronary heart disease, and heart attack. The age category 61–70 years showed the highest prevalence of angina pectoris and stroke. Figure 2 shows the frequency distribution of the cumulative number of chronic comorbid conditions in the different age groups, which gradually increased with age. The majority of the study subjects in the age categories up to 50 years had two or less other chronic conditions, while in the two oldest age categories approximately 65% of subjects suffered from three or more.

**Table 2:** Prevalence of the investigated chronic comorbid conditions in the respective asthma exacerbation categories.

	Asthma exacerbation category			
	No exacerbations *n* = 1344	Moderate exacerbations *n* = 762	Severe exacerbations *n* = 281	Total *n* = 2387
Obesity^a^	609 (47.4)	363 (49.7)	163 (59.5)	1135 (49.6)
Depression	431 (32.1)	276 (36.2)	106 (37.7)	813 (34.1)
Hypertension	423 (31.5)	218 (28.6)	83 (29.5)	724 (30.3)
Hypercholesterolemia	339 (25.2)	172 (22.6)	59 (21.0)	570 (23.9)
Diabetes mellitus	207 (15.4)	111 (14.6)	55 (19.6)	373 (15.6)
GERS^b^	92 (12.7)	61 (16.4)	22 (16.5)	175 (14.2)
Rheumatoid arthritis	169 (12.6)	116 (15.2)	45 (16.0)	330 (13.8)
Current soft drug use^c^	154 (11.5)	87 (11.4)	32 (11.4)	273 (11.4)
Thyroid problems	122 (9.1)	86 (11.3)	27 (9.6)	235 (9.8)
Gout	72 (5.4)	42 (5.5)	10 (3.6)	124 (5.2)
History of heart attack	62 (4.6)	29 (3.8)	20 (7.1)	111 (4.7)
History of stroke	51 (3.8)	46 (6.0)	16 (5.7)	113 (4.7)
Kidney failure	56 (4.2)	32 (4.2)	16 (5.7)	104 (4.4)
Congestive heart failure	43 (3.2)	31 (4.1)	16 (5.7)	90 (3.8)
Angina pectoris	43 (3.2)	26 (3.4)	16 (5.7)	85 (3.6)
Current hard drug use^d^	41 (3.1)	28 (3.7)	10 (3.6)	79 (3.3)
Coronary heart disease	45 (3.3)	20 (2.6)	9 (3.2)	74 (3.1)
Liver condition	36 (2.7)	24 (3.1)	7 (2.5)	67 (2.8)

Comorbid conditions are sorted based on the prevalence in the total study population. For each category *n* (%) are shown.

*BMI* Body Mass Index, *GERS* gastroesophageal reflux-like symptoms.

^a^ BMI ≥ 30 kg/m^2^.

^b^ GERS survey years 2013–2014, 2015–2016, and 2017–2018 (*n* = 1232).

^c^ Marijuana or hashish use in the last year.

^d^ Cocaine, heroin or methamphetamine use in the last year.

### Chronic comorbidities associated with asthma exacerbation occurrence

Table 3 shows the results of the univariable and multivariable ordinal regression analyses for the associations between the respective chronic comorbid conditions and asthma exacerbation category. The multivariable analyses showed the following chronic comorbid conditions to be associated with an increased odds of having severe asthma exacerbations: obesity (OR = 1.67, 95% CI 1.24 to 2.26) and rheumatoid arthritis (OR = 1.55, 95% CI 1.04 to 2.30). Conditions associated with increased odds of moderate asthma exacerbations were history of stroke (OR = 1.95, 95% CI 1.22 to 3.11) and rheumatoid arthritis (OR = 1.33, 95% CI 1.00 to 1.75).

**Table 3:** Results from univariable and multivariable ordinal regression models for each of the chronic comorbid conditions on asthma exacerbation occurrence.

	Asthma exacerbation category							
	Moderate exacerbations vs. no exacerbations				Severe exacerbations vs. no exacerbations			
	Univariable analysis		Multivariable analysis^a^		Univariable analysis		Multivariable analysis^a^	
	Crude OR (95% CI)	*p*-value	Adjusted OR (95% CI)	*p*-value	Crude OR (95% CI)	*p*-value	Adjusted OR (95% CI)	*p*-value
Obesity^b^	1.10 (0.91, 1.31)	0.328	1.00 (0.99, 1.00)	0.103	1.63 (1.25, 2.12)	<0.001	**1.67 (1.24, 2.26)**	**0.001**
Depression	1.20 (1.00, 1.45)	0.053	1.17 (0.95, 1.43)	0.142	1.28 (0.98, 1.68)	0.067	1.16 (0.86, 1.55)	0.337
Hypertension	0.87 (0.72, 1.06)	0.170	0.81 (0.62, 1.06)	0.131	0.91 (0.69, 1.21)	0.524	0.72 (0.48, 1.07)	0.100
Hypercholesterolemia	0.86 (0.70, 1.07)	0.173	0.91 (0.70, 1.18)	0.473	0.79 (0.58, 1.08)	0.135	0.81 (0.55, 1.20)	0.296
Diabetes mellitus	0.94 (0.73, 1.20)	0.607	1.01 (0.76, 1.35)	0.921	1.34 (0.96, 1.86)	0.085	1.27 (0.85, 1.88)	0.240
GERS^c^	1.35 (0.95, 1.92)	0.091	1.11 (0.73, 1.71)	0.624	1.37 (0.82, 2.27)	0.226	1.24 (0.66, 2.32)	0.511
Rheumatoid arthritis	1.25 (0.97, 1.61)	0.088	**1.33 (1.00, 1.75)**	**0.048**	1.33 (0.93, 1.90)	0.122	**1.55 (1.04, 2.30)**	**0.032**
Soft drug use^d^	1.00 (0.75, 1.32)	0.996	1.01 (0.72, 1.41)	0.972	0.99 (0.66, 1.49)	0.973	0.89 (0.55, 1.43)	0.620
Thyroid problems	1.27 (0.95, 1.71)	0.103	1.07 (0.76, 1.50)	0.704	1.07 (0.69, 1.65)	0.779	0.99 (0.59, 1.64)	0.958
Gout	1.03 (0.70, 1.52)	0.880	1.07 (0.67, 1.70)	0.775	0.65 (0.33, 1.28)	0.214	0.59 (0.28, 1.25)	0.165
History of heart attack	0.82 (0.52, 1.28)	0.382	0.95 (0.53, 1.69)	0.861	1.58 (0.94, 2.67)	0.084	1.80 (0.89, 3.67)	0.103
History of stroke	1.63 (1.09, 2.45)	0.019	**1.95 (1.22, 3.11)**	**0.005**	1.53 (0.86, 2.73)	0.148	1.67 (0.86, 3.21)	0.128
Kidney failure	1.01 (0.65, 1.57)	0.971	0.96 (0.57, 1.61)	0.879	1.39 (0.78, 2.46)	0.260	1.21 (0.61, 2.43)	0.586
Congestive heart failure	1.28 (0.80, 2.05)	0.299	1.36 (0.76, 2.43)	0.307	1.83 (1.01, 3.29)	0.045	1.45 (0.69, 3.04)	0.330
Angina pectoris	1.07 (0.65, 1.75)	0.792	0.87 (0.48, 1.57)	0.639	1.83 (1.01, 3.29)	0.045	0.98 (0.45, 2.13)	0.955
Hard drug use^e^	1.21 (0.74, 1.98)	0.440	1.16 (0.67, 2.01)	0.591	1.17 (0.58, 2.37)	0.657	1.08 (0.50, 2.36)	0.839
Coronary heart disease	0.78 (0.46, 1.33)	0.778	0.75 (0.36, 1.56)	0.440	0.96 (0.46, 1.98)	0.902	0.96 (0.36, 2.57)	0.936
Liver condition^f^	—	—	—	—	—	—	—	—

Comparisons shown are for moderate *versus* no exacerbations and for severe versus no exacerbations categories. Comorbid conditions are sorted based on the prevalence in the total study population. Crude and adjusted odds ratios and 95% confidence intervals are shown. Statistically significant associations are marked in **bold** typeface.

*BMI* Body Mass Index, *CI* confidence interval, *GERS* gastroesophageal reflux-like symptoms, *OR* odds ratio.

^a^ Multivariable models corrected for sex; race; age at screening; time since asthma diagnosis; health insurance coverage; poverty income ratio; cigarette smoking status; and asthma-related medication if appropriate (i.e., *p* ≤ 0.1 in the final model for each comorbid condition).

^b^ BMI ≥ 30 kg/m^2^.

^c^ GERS survey years 2013–2014, 2015–2016, and 2017–2018.

^d^ Marijuana or hashish use in the past year.

^e^ Cocaine, heroin or methamphetamine use in the past year.

^f^ Comorbid conditions with counts below 8 subjects in one or more asthma exacerbation categories were not statistically tested.

Table 4 shows the odds ratios from the univariable and multivariable ordinal regression analyses split in the different age categories. In the youngest age category (18–30 years) soft drug use was associated with a decreased odds of severe exacerbations (OR = 0.40, 95% CI 0.17, 0.97). In subjects aged 31–40 years obesity (OR = 2.01, 95% CI 1.15 to 3.53) and depression (OR = 2.25, 95% CI 1.32, 3.81) were associated with increased odds of moderate exacerbations. In subjects aged 41 to 50 years obesity was associated with increased odds of severe asthma exacerbations (OR = 3.07, 95% CI 1.31 to 7.19), whereas in those aged 51 to 60 years or 61 to 70 years this was true for thyroid problems (OR = 4.49, 95% CI 1.69 to 11.95) and rheumatoid arthritis (OR = 2.39, 95% CI 1.04 to 5.47), respectively. In subjects aged 71 years or older no comorbid conditions were found to be associated with moderate or severe exacerbations.

**Table 4:** Statistically significant odds ratios from the univariable and multivariable ordinal regression models for associations between the investigated chronic comorbid conditions and asthma exacerbation occurrence in the consecutive age categories.

		Asthma exacerbation category							
		Moderate exacerbations vs. no exacerbations				Severe exacerbations vs. no exacerbations			
		Univariable analysis		Multivariablete analysis^a^		Univariable analysis		Multivariable analysis^a^	
Age category (years)	Chronic comorbid condition	Crude OR (95% CI)	*p*-value	Adjusted OR (95% CI)	*p*-value	Crude OR (95% CI)	*p*-value	Adjusted OR (95% CI)	*p*-value
18–30	Soft drug use^b^	0.78 (0.51, 1.18)	0.240	0.71 (0.42, 1.19)	0.197	0.57 (0.30, 1.10)	0.092	**0.40 (0.17, 0.97)**	**0.042**
31–40	Obesity^c^	1.68 (1.06, 2.67)	0.028	**2.01 (1.15, 3.53)**	**0.014**	1.63 (0.87, 3.04)	0.127	1.89 (0.93, 3.84)	0.090
	Depression	1.82 (1.15, 2.89)	0.011	**2.25 (1.32, 3.81)**	**0.003**	1.45 (0.78, 2.71)	0.239	1.58 (0.81, 3.11)	0.183
41–50	Obesity	1.11 (0.70, 1.73)	0.664	1.28 (0.76, 2.14)	0.361	2.94 (1.39, 6.22)	0.005	**3.07 (1.31, 7.19)**	**0.010**
51–60	Thyroid problems	2.30 (1.18, 4.47)	0.015	1.78 (0.81, 3.90)	0.150	3.25 (1.46, 7.21)	0.004	**4.49 (1.69, 11.95)**	**0.003**
61–70	Rheumatoid arthritis	1.43 (0.84, 2.42)	0.186	1.32 (0.76, 2.31)	0.324	2.60 (1.22, 5.55)	0.013	**2.39 (1.04, 5.47)**	**0.039**
≥71	—	—	—	—	—	—	—	—	—

Crude and adjusted odds ratios and 95% confidence intervals are shown. Statistically significant associations are marked in **bold** typeface.

*BMI* Body Mass Index, *CI* confidence interval, *OR* odds ratio.

^a^ Models corrected for sex; race; time since asthma diagnosis; health insurance coverage; poverty income ratio; cigarette smoking status; and asthma-related medication if appropriate (i.e., *p* ≤ 0.1 in the final model for each comorbid condition). Variables with counts below 8 were not statistically tested.

^b^ Marijuana or hashish use in the past year.

^c^ BMI ≥ 30 kg/m^2^.

## Discussion

In this retrospective cohort study based on the NHANES program we explored associations between chronic comorbid conditions and the occurrence of moderate and severe exacerbations in adult subjects with a self-reported diagnosis of asthma drawn from the US general population. We found that obesity and rheumatoid arthritis were statistically significant associated with the odds of severe asthma exacerbations and that rheumatoid arthritis was associated with the odds of moderate asthma exacerbations, as was a history of stroke. Stratification of the study sample in age categories revealed specific associations between certain comorbid conditions and asthma exacerbation occurrence. For example, soft drug use was especially high in younger subjects (i.e., those aged between 18 and 30 years) and associated with a reduced occurrence of severe exacerbations, while depression was associated with an increased occurrence of moderate asthma exacerbations in subjects aged 31–40 years.

A link between obesity and risk of asthma exacerbations has been reported in several previous studies^6–8,18^. Our observed association between rheumatoid arthritis and (moderate as well as severe) asthma exacerbations is consistent with a recent study from Luo et al.^15^. A possible mechanism could be that patients with rheumatoid arthritis have a dysregulated immune system and often receive treatment with immunosuppressive medication, which may lead to a higher risk of acute respiratory infections (ARI)^19^, a known trigger for asthma exacerbations^2^. The observed association in our study of a history of stroke and increased asthma exacerbation occurrence is again in line with previous studies^13,14,18^. Underlying mechanisms may be that asthma patients with a history of stroke are not only more prone to ARI leading to asthma exacerbation, but also to systemic vascular inflammation with platelet activation, inhibition of fibrinolysis, and elevation of C-reactive protein levels resulting in cardiovascular events^20^. Besides, increased stroke risk may also be the result of increased atrial fibrillation risk in uncontrolled asthma^11^, as a result of asthma exacerbation management with β_2_-agonists, discontinuation of β-blockers and discontinuation of aspirin in patients with aspirin-exacerbated respiratory disease^14^. In our study, a history of stroke was associated with moderate exacerbations, but not with severe exacerbations. The limited number of subjects in the severe exacerbations category and thus a Type 2 error may explain this.

In the age category of 31–40 years, we found that symptoms of depression were associated with moderate asthma exacerbations. A meta-analysis based on prospective cohort studies strongly suggests that depression significantly increased the risk of asthma exacerbation^16^. However, we did not observe this association in the total study population nor in the other age categories. Possible explanations for this are that we used a questionnaire-based (PHQ-9) definition for depressive symptoms in the past two weeks instead of a clinical diagnosis of depression, or that previous studies have mainly included asthma patients in this particular age range. Conceivable mechanisms underlying the interaction between depression and asthma exacerbation are that depression may accompany behavior changes in terms of less help-seeking and non-adherence to medication but may also contribute to risk behaviors resulting in smoking, physical inactivity, and obesity^16^. Furthermore, cerebral changes in depression play a possible role in asthma symptoms^21^ and long-term stress stimulation may influence airway inflammation and asthma severity^17^.We also observed a statistically significant association between thyroid problems and severe asthma exacerbations in subjects aged between 51 and 60 years. This observation is difficult to interpret since we cannot determine, based on the data available, what the specific underlying thyroid problems were. Nonetheless, a recent study has reported a close relationship between thyroid hormone levels and severity of asthma in older adults^22^, providing a possible explanation for the observed association in our study.

In our study current soft drug use in the 18 to 30 years age group showed a statistically significant association with a decreased occurrence of severe asthma exacerbations. This is in contrast with a 2017 review, which found no or insufficient evidence for a statistical association between cannabis smoking and asthma exacerbation^23^. One possible explanation for our deviating finding could be that younger subjects who have sufficiently controlled asthma may be more likely to smoke cannabis or marijuana than those whose asthma is less well controlled. Another explanation might be that we did not include the most prevalent allergic disease in the younger population (allergic rhinitis and atopic dermatitis) in the study.

A clear strength of our study is that we investigated a large sample of 2387 subjects with doctor-diagnosed asthma in a study population that is presumably representative for the asthma population in the United States. The data we used is from the well-established NHANES program, which routinely collects data every two years using validated measures and questionnaire administration by trained medical personnel^24^. The data collection in NHANES is rather extensive and robust, which guarantees high-quality data regarding participants’ asthma exacerbations, comorbid conditions, smoking habits, and other risk factors for asthma exacerbations (e.g., PIR and asthma preventer medication use). We also consider the differentiation between moderate and severe exacerbations and the age-stratified analysis of chronic comorbid conditions in relation to asthma exacerbation occurrence strengths of our study, as this provides a more detailed insight compared to pooling all exacerbations and all age groups together.

On the other hand, the main limitation is that this cross-sectional study is depending on self-report of participants, which may have caused self-reporting bias to occur. For instance, 228 participants answered affirmatively on the question about once having received a diagnosis of chronic bronchitis, but that the diagnosis is not active anymore. These participants were not excluded from the study, but incorrect affirmative answers may have led to some subjects who also suffered from COPD (i.e., asthma-COPD overlap) to be included in the study sample. Ideally all NHANES participants would have had an extensive respiratory assessment to establish whether or not asthma (and/or COPD) was present^25^. Besides, the most reflective definition for moderate and severe asthma exacerbations could not be derived from the data: an episode of progressive increase in asthma symptoms that required a change in treatment^2^, with ideally health-record derived additional information about the medical management and the in- or outpatient setting during this episode. Thirty-one participants denied having had an episode of asthma worsening in the previous year, but at the time same indicated to have visited an emergency room due to their asthma. We categorized these subjects in the severe asthma exacerbation category, but misclassification may have occurred as the actual validity of the NHANES survey questions on occurrence of exacerbations (i.e., *‘During the past 12 months, have you had an episode of asthma or an asthma attack?’* and *‘During the past 12 months, have you had to visit an emergency room (ER) or urgent care center because of asthma?’*) may be limited and phrasing these questions as asthma being ‘worse’ or ‘out of control’ instead may be a better option^26^.

No information to verify the chronic nature of certain comorbid conditions (e.g., gout and depression) could be extracted from the data, leading to the possible inclusion of single episodes of the conditions instead of truly chronic conditions. The use of a PHQ-9-based definition for depressive symptoms as a surrogate for clinical depression is also a limitation, as a questionnaire-based definition and an actual clinical diagnosis of depression do not necessarily coincide^27^. Finally, a notable observation was that ICS were used by a relatively small proportion of the study sample (13.6%), which is quite a bit lower than the 30.8% in the CDC’s nationwide BRFSS Asthma Call-back Survey in active asthma^28^. Although questionnaire-based self-reports of asthma diagnoses are widely used in population surveys^29,30^, the relatively low rate of ICS use does raise some concern about the validity of participants’ asthma diagnoses in the NHANES survey data. On the other hand inadequate asthma therapy among adults has been demonstrated to be an important issue in the US^31^ and elsewhere^32^. The high use of SABA combined with the low use of ICS by subjects in the moderate and severe exacerbations categories fits the observed increased exacerbation occurrence^33^.

It would have been interesting to determine whether allergic rhinitis, chronic sinusitis, atopic dermatitis and obstructive sleep apnoea are also associated with asthma exacerbations, but data on these conditions is not available in the NHANES database. A final limitation is that there was no measure to determine asthma control available, like—for instance—the Asthma Control Test (ACT)^34^.

In conclusion, in this study obesity and rheumatoid arthritis were associated with the occurrence of severe asthma exacerbations, whereas rheumatoid arthritis and a history of stroke were associated with the occurrence of moderate asthma exacerbations. Age-stratified analysis showed soft drug use, obesity, depression, thyroid problems, and rheumatoid arthritis to be associated with moderate and/or severe exacerbation occurrence in one or more 10-year age strata. These findings confirm but also complement the current body of knowledge about the role that specific chronic comorbid conditions may have in exacerbation-prone asthma. Health professionals involved in asthma management should be aware of other chronic conditions their asthma patients may have and incorporate this information in their exacerbation risk estimation, chronic disease management, and personalized asthma care.

## Methods

### Study design and study population

We performed a cross-sectional study nested in a cohort study based on the 2007–2018 data from the two-yearly National Health and Nutrition Examination Survey (NHANES), a program of studies from the US National Center for Health Statistics designed to assess the health and nutritional status of adults and children in the United States^24^. The survey comprises an extensive interview questionnaire on demographic and health-related issues and a range of physical examinations (physician’s exam, height, weight, and other body measures; blood pressure measurement; bone density measurement; liver ultrasound; lab tests on blood and urine, among other clinical assessments). The questionnaire and measurements are administered by trained medical personnel. Random selection of participants aims to produce a representative study sample that reflects the US population^24^.

**Fig. 3:**
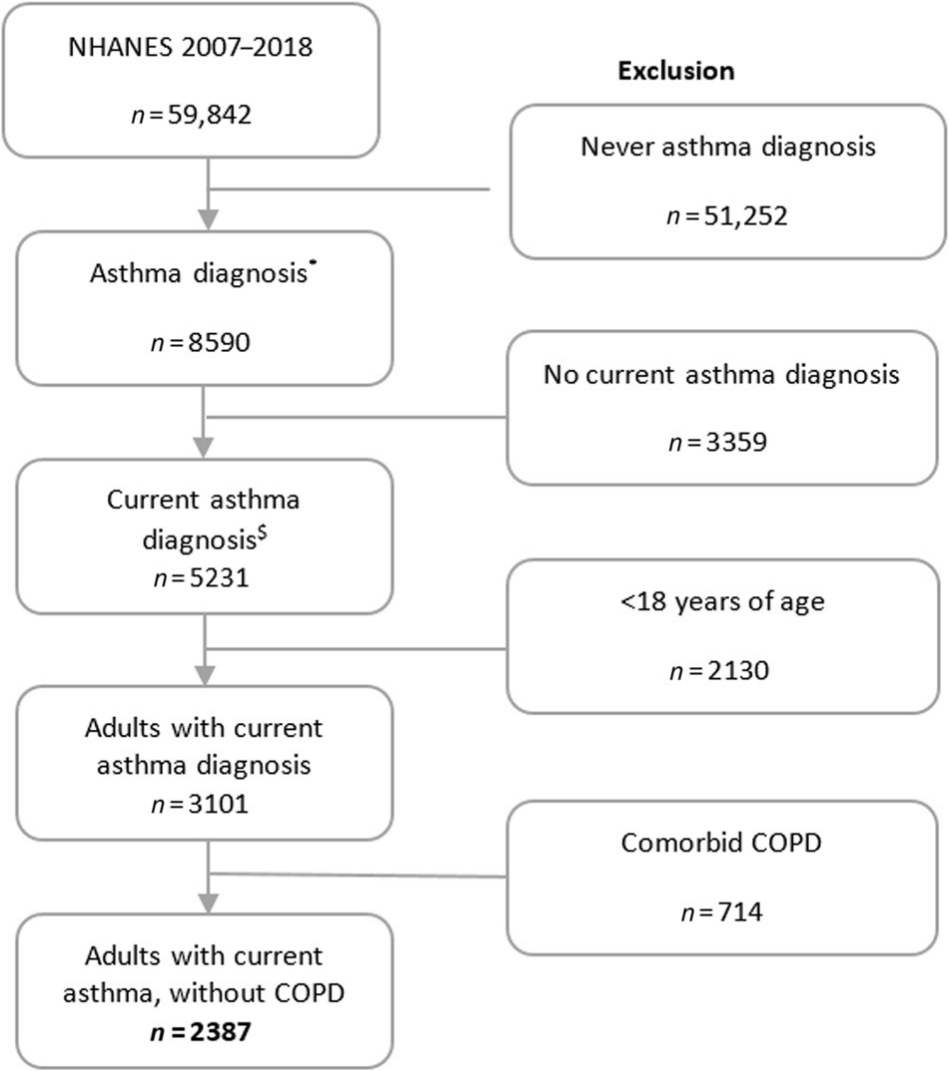
Flowchart of the selection of study subjects from the biennial NHANES survey cohorts. COPD chronic obstructive pulmonary disease. NHANES National Health and Nutrition Examination Survey. * self-reported asthma diagnosis based on question ‘Has a doctor ever told you that you have asthma?’ ^$^ current asthma diagnosis based on question ‘Do you still have asthma?’.

From the consecutive NHANES datasets we selected individuals with a self-reported diagnosis of asthma using the question: *‘Has a doctor ever told you that you have asthma?’* (*n* = 8590; Fig. 3). From this group we selected subjects aged 18 years or older with an affirmative response to the question *‘Do you still have asthma?’*. Subjects who reported to have received a healthcare provider-diagnosis of chronic obstructive pulmonary disease (COPD) or emphysema or reported that they still had chronic bronchitis were excluded because these respiratory conditions are also accompanied by exacerbations. The final study population consisted of 2387 adults with a self-reported asthma diagnosis.

All NHANES survey cycles are approved by the National Center for Health Statistics Research Ethics Review Board. The protocols of the 2007 until 2018 surveys are available at https://www.cdc.gov/nchs/nhanes/irba98.htm. All participants provided informed consent.

### Asthma exacerbation categories

The study population was divided into three ‘asthma exacerbation categories’, based on two questions: (i) *‘During the past 12 months, have you had an episode of asthma or an asthma attack?’* and (ii) *‘During the past 12 months, have you had to visit an emergency room (ER) or urgent care center because of asthma?’*. Study subjects with negative replies to both questions were classified as ‘no asthma exacerbation’, a positive answer to question (i) and a negative answer to question (ii) as ‘moderate asthma exacerbation’, and a positive answer to question (ii) as ‘severe asthma exacerbation’ (i.e., independent of the reply to question (i)). These three asthma exacerbation categories constituted the single response variable for the statistical analysis.

### Chronic comorbidities

The NHANES data allowed us to look at eighteen chronic comorbid conditions: angina pectoris; congestive heart failure; coronary heart disease; diabetes mellitus; gastroesophageal reflux-like symptoms (GERS); gout; history of heart attacks; history of stroke; hypercholesterolemia; hypertension; kidney failure; liver disease; obesity; rheumatoid arthritis; and thyroid problems (see Supplementary Table 1 for operationalization). We defined these conditions as being chronic based on their nonreversible nature and/or the increased lifetime risk of occurrence of recurrent episodes. Because depression, soft drug (i.e., marijuana or hashish) use and hard drug (cocaine, heroin, or methamphetamine) use can be recurrent we decided to consider these as chronic conditions too.

Depression was assessed using the Diagnostic and Statistical Manual of Mental Disorders, fourth edition (DSM-IV) validated Patient Health Questionnaire-9 (PHQ-9), a screening instrument for frequency of depressive symptoms over the past two weeks. Depression was defined as ‘mild depression’ (PHQ-9 score 5 to 9), or ‘moderate to severe depression’ (PHQ-9 score ≥10)^35^.

Gastroesophageal reflux-like symptoms (GERS) were defined using International Classification of Diseases, Tenth Revision, Clinical Modification (ICD-10-CM) codes from study subjects’ prescription medication use in the last month prior to the survey: ‘heartburn’ (R12), ‘gastro-esophageal reflux disease’ (K21), ‘gastric ulcer’ (K25), ‘peptic ulcer, site unspecified’ (K27), and ‘functional dyspepsia’ (K30). These data were available for three survey cycles (2013–2014, 2015–2016, and 2017–2018).

Obesity was defined as Body Mass Index (BMI) ≥ 30 kg/m^2^.

### Demographic, smoking, and asthma treatment data

We also extracted the following information from the NHANES survey data for the included study subjects: sex; age at the time of screening; race; time since asthma diagnosis; health insurance coverage; poverty income ratio; asthma-related medication; and cigarette smoking status, the latter categorized as current, former, and never smoker. A subject was considered a current smoker when (s)he reported active smoking in the year prior to the survey and having smoked at least 100 cigarettes in his/her lifetime. Poverty to income ratio (PIR), an indicator of socioeconomic status, divides family income by the poverty threshold. PIR was categorized as income at or below the poverty line ( ≤ 1) or above the poverty line ( >1)^36^.

We identified the following asthma-related prescription medication use in the past month: inhaled corticosteroids (ICS); short-acting β2-agonists (SABA); long-acting β2-agonists (LABA); anticholinergics; antihistamines; leukotriene modifiers; oral corticosteroids (OCS); and other (i.e., cromolyn, epinephrine, immunomodulator medication, phosphodiesterase-4 (PDE_4_) inhibitors, and unspecified anti-asthmatic combinations, -respiratory agents, and -respiratory inhalant products).

### Statistical analysis

We compared baseline characteristics between the ‘no asthma exacerbation’, ‘moderate asthma exacerbation’, and ‘severe asthma exacerbation’ categories using one-way ANOVA for normally distributed, and Kruskal-Wallis tests for nonnormally distributed continuous variables. Chi-square and Fisher’s exact tests were used to compare categorical variables. Variables with counts below eight in one or more exacerbation categories were not statistically tested. No correction for multiple comparisons was applied. We calculated prevalence rates for the eighteen chronic comorbid conditions studied and the occurrence of the total number of chronic comorbid conditions across age categories.

To analyze associations between each separate comorbid condition and asthma exacerbation categories (the response variable) we used univariable and multivariable ordinal logistic regression models to estimate odds ratios for the moderate exacerbations category relative to the no exacerbations category, and for the severe exacerbations category relative to the no exacerbations category. For the multivariable analysis we started with a full model with all available potential confounders. Backward elimination was used to drop covariates with *p* > 0.1 from the ordinal logistic regression model. We consider the final (i.e., reduced) multivariable models to be the main results of the analysis. All statistical analyses were performed using SPSS statistical software (IBM Corp. Released 2017. IBM SPSS Statistics for Windows, version 25.0. Armonk, NY: IBM Corp). A two-sided p-value below 0.05 was considered statistically significant.

### Reporting summary

Further information on research design is available in the Nature Research Reporting Summary linked to this article.

### Supplementary information

Supplementary Material

Reporting Summary

## Data Availability

The data supporting the results reported in the article can be found at https://wwwn.cdc.gov/nchs/nhanes/. The SPSS syntax files used to analyze to dataset are available on request to the corresponding author.
