# Supplementary material for: Chronic comorbid conditions and asthma exacerbation occurrence in a general population sample
Source: NPJ Prim Care Respir Med. 2023 Aug 11;33:29. doi: 10.1038/s41533-023-00350-x (PMC10421910; doi:10.1038/s41533-023-00350-x)
Supplement: Supplementary file 1 — Supplementary Material [file 41533_2023_350_MOESM1_ESM.pdf]

Supplementary Table 1. Operationalization of chronic comorbidity based on the National Health and Nutrition Examination Survey (NHANES) data

	**Chronic condition**	**NHANES data file name**	**NHANES data file**	**Question(s)/item(s) used for analyses (response)**
Systemic diseases	Obesity	Body Measures (BMX_J)	Examination data	Body Mass Index (kg/m2)
	Rheumatoid arthritis	Medical Conditions (MCQ_J)	Questionnaire data	Doctor ever told that you had arthritis? (Yes)<br>Which type of arthritis was it?<br>• Osteoarthritis or degenerative arthritis<br>• Rheumatoid arthritis (Yes)<br>• Psoriatic arthritis<br>• Other
	Gout	Medical Conditions (MCQ_J)	Questionnaire data	Doctor ever told that you had gout? (Yes)
Cardiovascular diseases	Congestive heart failure	Medical Conditions (MCQ_J)	Questionnaire data	Doctor ever told you had congestive heart failure? (Yes)
	Coronary heart disease	Medical Conditions (MCQ_J)	Questionnaire data	Doctor ever told you had coronary heart disease? (Yes)
	Angina pectoris	Medical Conditions (MCQ_J)	Questionnaire data	Doctor ever told you had angina/angina pectoris? (Yes)
	Heart attack	Medical Conditions (MCQ_J)	Questionnaire data	Doctor ever told you had a heart attack? (Yes)
	Hypertension	Blood Pressure & Cholesterol (BPQ_J)	Questionnaire data	Now taking prescribed medicine for HBP? (Yes)
	Hypercholesterolemia	Blood Pressure & Cholesterol (BPQ_J)	Questionnaire data	To lower your blood cholesterol, have you ever been told by a doctor or other health professional to take prescribed medicine? (Yes)
	Stroke	Medical Conditions (MCQ_J)	Questionnaire data	Doctor ever told you had a stroke? (Yes)
Organ diseases	Liver condition	Medical Conditions (MCQ_J)	Questionnaire data	Doctor ever told you had any liver condition? (Yes)
	Liver condition	Medical Conditions (MCQ_J)	Questionnaire data	Do you still have a liver condition? (Yes)
	Kidney failing	Kidney Conditions - Urology (KIQ_U_J)	Questionnaire data	Doctor ever told you had weak/failing kidneys? (Yes)
	Thyroid problem	Medical Conditions (MCQ_J)	Questionnaire data	Doctor ever told you had thyroid problem? (Yes)
	Diabetes	Diabetes (DIQ_J)	Questionnaire data	Doctor ever told you have diabetes? (Yes)
Psychiatric conditions	Depression	Mental Health - Depression Screener (DPQ_J)	Questionnaire data	• Have little interest in doing things<br>• Feeling down, depressed, or hopeless<br>• Trouble sleeping or sleeping too much<br>• Feeling tired or having little energy<br>• Poor appetite or overeating<br>• Feeling bad about yourself<br>• Trouble concentrating on things<br>• Moving or speaking slowly or too fast<br>• Thought you would be better off dead<br>• Difficulty these problems have caused`<br>(sum score 5 to 10 is Yes, sum score 0 to 4 is No)
Other	Gastroesophageal Reflux Like Symptoms (GERS)	Prescription Medications (RXQ_RX_J)	Questionnaire data	Reason for use (as reported by participant) data release variables:<br>Heartburn (R12) and/or gastro-esophageal reflux disease (K21) and/or gastric ulcer (K25) and/or peptic ulcer and/or site unspecified (K27) and/or functional dyspepsia (K30) is Yes
	Current soft drug use	Drug Use (DUQ_J)	Questionnaire data	How long has it been since you last smoked marijuana or hashish at least once a month for one year? (Days, Weeks, or Months is Yes)
	Current hard drug use	Drug Use (DUQ_J)		How long has it been since you last used cocaine, in any form? (Days, Weeks, or Months is Yes)
		Drug Use (DUQ_J)		How long has it been since you last used heroin? (Days, Weeks, or Months is Yes)
		Drug Use (DUQ_J)		How long has it been since you last used methamphetamine? (Days, Weeks, or Months is Yes)
